# TNFα promotes proliferation of human synovial MSCs while maintaining chondrogenic potential

**DOI:** 10.1371/journal.pone.0177771

**Published:** 2017-05-18

**Authors:** Mikio Shioda, Takeshi Muneta, Kunikazu Tsuji, Mitsuru Mizuno, Keiichiro Komori, Hideyuki Koga, Ichiro Sekiya

**Affiliations:** 1 Department of Joint Surgery and Sports Medicine, Graduate School of Medicine, Tokyo Medical and Dental University, Tokyo, Japan; 2 Department of Cartilage Regeneration, Graduate School of Medicine, Tokyo Medical and Dental University, Tokyo, Japan; 3 Center for Stem Cell and Regenerative Medicine, Tokyo Medical and Dental University, Tokyo, Japan; University of Wisconsin-Madison, UNITED STATES

## Abstract

Synovial mesenchymal stem cells (MSCs) are a candidate cell source for cartilage and meniscus regeneration. If we can proliferate synovial MSCs more effectively, we can expand clinical applications to patients with large cartilage and meniscus lesions. TNFα is a pleiotropic cytokine that can affect the growth and differentiation of cells in the body. The purpose of this study was to examine the effect of TNFα on proliferation, chondrogenesis, and other properties of human synovial MSCs. Passage 1 human synovial MSCs from 2 donors were cultured with 2.5 x 10^−12^~10^−7^ g/ml, 10 fold dilution series of TNFα for 14 days, then the cell number and colony number was counted. The effect of the optimum dose of TNFα on proliferation was also examined in synovial MSCs from 6 donors. Chondrogenic potential of synovial MSCs pretreated with TNFα was evaluated in 6 donors. The expressions of 12 surface antigens were also examined in 3 donors.2.5 ng/ml and higher concentration of TNFα significantly increased cell number/dish and cell number/colony in both donors. The effect of 25 ng/ml TNFα was confirmed in all 6 donors. There was no significant difference in the weight, or amount of glycosaminoglycan and DNA of the cartilage pellets between the MSCs untreated and MSCs pretreated with 25 ng/ml TNFα. TNFα decreased expression rate of CD 105 and 140b in all 3 donors. TNFα promoted proliferation of synovial MSCs with increase of cell number/ colony. Pretreatment with TNFα did not affect chondrogenesis of synovial MSCs. However, TNFα affected some properties of synovial MSCs.

## Introduction

Synovial mesenchymal stem cells (MSCs) are a candidate cell source for regenerative medicine of cartilage and menisci due to their high chondrogenic ability [1–3]. In clinical situations, transplantation of autologous synovial MSCs for 10 patients with a symptomatic single cartilage lesion of the femoral condyle in the knee joints was effective in terms of MRI, qualitative histologic findings, and Lysholm score [4]. We are currently performing a clinical study to investigate whether transplantation of autologous synovial MSCs promotes meniscus healing after degenerative meniscus injury [5–7]. In these clinical studies, we cultured autologous synovial MSCs for 14 days and prepared about 50 million synovial MSCs for transplantation. If we can proliferate synovial MSCs more effectively, we can expand clinical applications to osteoarthritis with several large cartilage and meniscus lesions.

Tumor necrosis factor (TNF) α was first identified and characterized according to the ability to induce the regression of tumors in animals [8, 9]. TNFα is a pleiotropic cytokine that can affect the growth, differentiation, and metabolism of virtually every nucleated cell type in the body [10]. TNFα is involved in systemic inflammation and its expression is also associated with pathologies, such as rheumatoid arthritis [11, 12]. TNFα induces proliferation of synovial fibroblasts derived from rheumatoid arthritis [13]; however, it is unknown whether TNFα promotes proliferation of synovial MSCs.

The effect of TNFα on chondrogenesis still remains controversial. Wehling et al. reported that TNFα inhibited chondrogenesis of human bone marrow MSCs through NF-κB-dependent pathways [14]. Contrarily, Michal et al. showed that TNFα did not impair the chondrogenic differentiation of bone marrow MSCs [15]. If TNFα can promote proliferation of synovial MSCs while maintaining chondrogenic ability, TNFα will be useful for regenerative medicine for cartilage and menisci with synovial MSCs. The purpose of this study was to examine the effect of TNFα on proliferation, chondrogenesis, and other properties of human synovial MSCs.

## Materials and methods

The present study was approved by the Medical Research Ethics Committee of Tokyo Medical and Dental University (No.2121) and full written informed consent was obtained from all patients.

### *Human* synovial MSCs

Human synovial tissue was harvested during total knee arthroplasty from knee joints of 14 patients with osteoarthritis. Synovium was digested with 3 mg/ml collagenase (Sigma-Aldrich, St. Louis, MO) at 37°C for 3 hours. Then, nucleated cells were cultured at a clonal density in 150 cm^2^ culture dishes (Nunc, Rochester, NY) in 20 ml α-minimum essential medium (αMEM: Thermo Fisher Scientific, Inc., Waltham, MA) containing 10% fetal bovine serum (FBS: Thermo Fisher Scientific, Inc., Waltham, MA) and 1% antibiotic-antimycotic (Thermo Fisher Scientific) for 14 days.

### Effect of TNFα on proliferation of synovial MSCs

Passage 1 synovial MSCs derived from 2 donors were plated at 10^4^ cells/60cm^2^ on 8 dishes, and cultured with 2.5 x 10^−12^~10^−7^ g/ml, 10 fold dilution series of TNFα (R&D Systems, Minneapolis, MN) or without TNFα for 14 days. Four dishes were stained with crystal violet to count the total number of cell colonies. Only colonies greater than 2 mm in diameter or distinctly stained colonies were counted. Synovial MSCs were harvested from the other 4 dishes and cell number/dish was counted with a hemocytometer.

To calculate cell number/colony, cell number/dish were counted from 4 dishes (dish A, dish B, dish C, dish D), and colony number/dish were counted from 4 additional dishes (dish E dish F, dish G, dish H). To calculate cell number/colony, cell number of dish A/colony number of dish E, cell number of dish B/colony number of dish F, cell number of dish C/colony number of dish G, and cell number of dish D/colony number of dish H were calculated respectively. Then average and standard deviation in cell number/colony (n = 4) were determined.

Passage 1 synovial MSCs derived from 6 donors were also plated at 10^4^ cells/60cm^2^ dish on 8 dishes, and cultured with 25 ng/ml TNFα or without TNFα for 14 days. The colony number/dish, cell number/dish, and cell number/colony were analyzed similarly.

### Differentiation

Passage 1 synovial MSCs derived from 6 donors were plated at 10^4^ cells/60cm^2^ with 25 ng/ml TNFα or without TNFα and cultured for 14 days. Then, 250,000 synovial MSCs pretreated with or without TNFα were placed in a 15 ml polypropylene tube (Becton Dickinson, Franklin lakes, NJ) and centrifuged at 450 x g for 10 minutes. The pellets were cultured for 21 days in 400 μl chondrogenic medium including 1μg/ml BMP-7 (OP-1, Stryker Biotech, Hopkinton, MA), 10 ng/ml transforming growth factor-β3 (R&D Systems), 100nM dexamethasone, 50 ng/ml ascorbate-2-phosphate, 40μl/ml proline, 100μl/ml pyruvate, and 50 mg/ml ITS + Premix (Becton Dickinson). The size, weight glycosaminoglycan (GAG) and DNA of the pellets were evaluated. Each pellet was digested in 1 ml papain buffer at 60°C for 24 hours. Fifty ul of the supernatant was added to 500 μl Blyscan Dye Regent (Blyscan Glycosaminoglycan Assay Kit: Biocolor, Carrickfergus, UK) and reacted on a mechanical shaker for 30 minutes. The unbound dye solution was removed by carefully inverting and draining the tubes. Five hundred μl dissociation reagent was added and the bound dye was released into the solution. Two hundred μl aliquots of each sample was transferred to the wells of a 96 well plate. The absorbance was measured by spectrophotometer at 656 nm. Each pellet was digested in 1 ml papain buffer at 60°C for 24 hours. Genomic DNA was extracted using phenol-chloroform (Thermo Fisher Scientific, Waltham, MA) and quantitated by microplate reader absorbance at 260 nm. The pellets were also evaluated histologically by safranin-o staining.

For adipogenesis and calcification, passage 1 synovial MSCs were plated at 10^4^ cells/60cm^2^ with 25 ng/ml TNFα or without TNFα and cultured for 14 days. Then, those cells were cultured in the adipogenic medium containing 0.5mM isobutyl-methylxanthine (Sigma-Aldrich), 100nM dexamethasone, 50mM indomethacin (Wako, Tokyo, Japan), calcification medium containing 1nM dexamethasone, 20mM β-glycerol phosphate (Wako), and 50mg/mL ascorbate-2-phosphate without TNFα for 21 days. The dishes were stained with 0.003% oil red-o solution for adipogenesis and with 0.5% alizarin red solution for calcification.

### Surface proteins

Passage 1 synovial MSCs of 3 patients were plated at 10^4^ cells/60cm^2^ dish and maintained in the presence or absence of 25 ng/ml TNFα for 14 days. MSC-related surface antigen expression was investigated by a flow cytometer (FACS Verse, BD Bioscience, CA). Ten million passage 2 cells were suspended and incubated in 50 μl of staining buffer (PBS supplemented with 2% FBS and 5mM EDTA) containing antibodies against cell surface antigens for 30 minutes at 4°C. Cells were rinsed and suspended in 0.7 ml of staining buffer for analysis. Data were analyzed by FACSuite software (BD Bioscience). The analysis was performed on samples from 3 donors. The list of antibodies employed in this experiment is displayed in S1 Table.

### Statistical analysis

Statcel 3 (OMS publishing Inc, Saitama, Japan) was used for statistical analyses. Analyses of variance (ANOVA) followed by the Tukey-Kramer test was used for assessing the effect of the different concentrations of TNFα on proliferation of synovial MSCs. The Wilcoxon signed-ranks test was used for assessing the effect of 25 ng/ml TNFα on proliferation of synovial MSCs and chondrogenesis.

## Results

### Effect of the different concentrations of TNFα on proliferation of synovial MSCs

**Fig 1:**
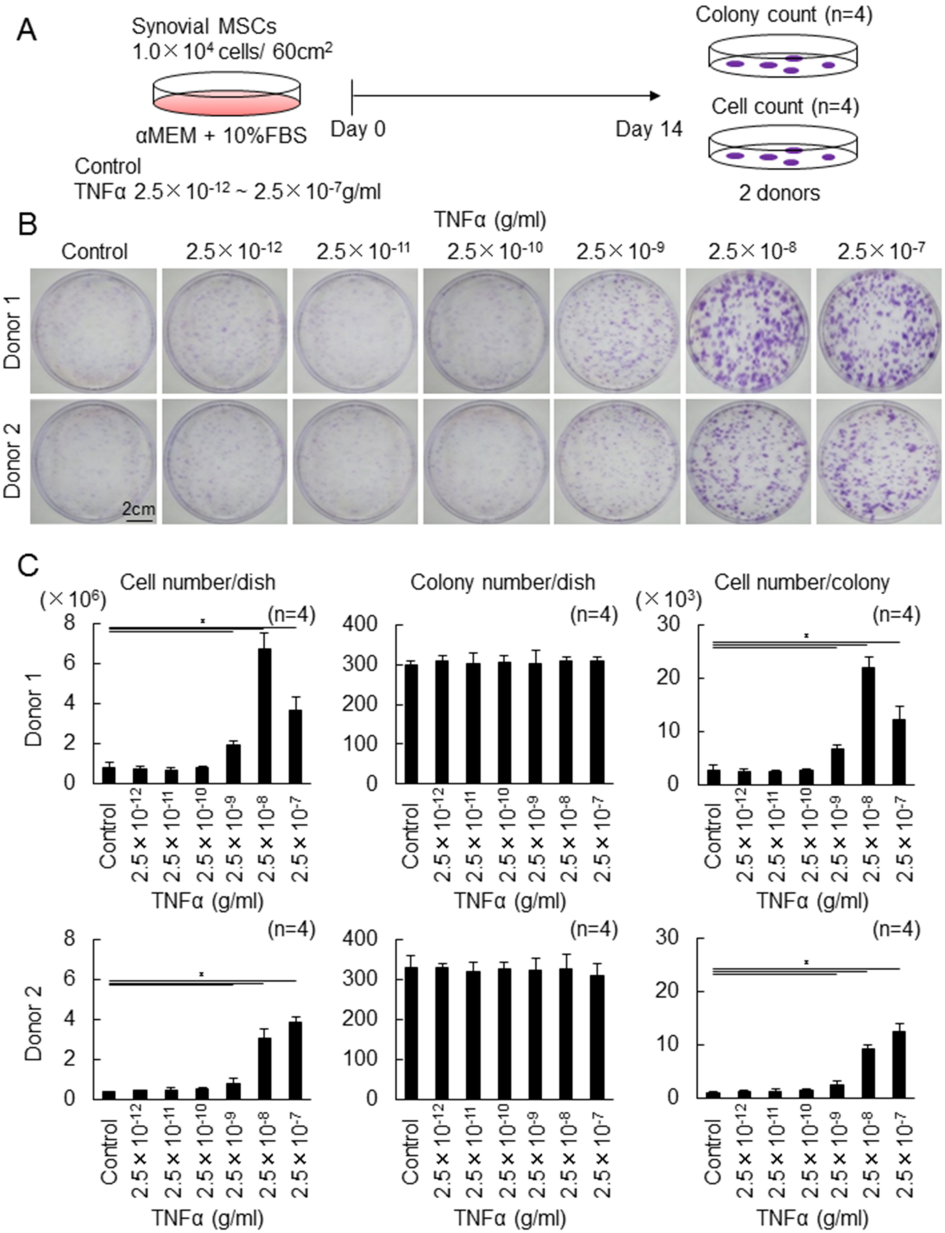
The effect of the different concentrations of TNFα on proliferation of synovial MSCs. (A) Experimental design. Passage 1 human synovial MSCs were plated at 10^4^ cells/60cm^2^ dish and cultured with the different concentrations of TNFα for 14 days. Colony number was counted from 4 dishes stained with crystal violet and cell number was measured after harvesting from other 4 dishes. (B) Representative dishes stained with crystal violet (2 donors). (C) Cell number/dish, colony number/dish, and cell number/colony. Average values with standard derivation are shown (*p<0.05 by Tukey-Kramer test).

First of all, the effect of the different concentrations of TNFα on colony formation and proliferation of synovial MSCs was examined (Fig 1A). Apparently, the addition of TNFα made colony formation more obviously dose-dependent in 2 donors (Fig 1B). Quantification analyses demonstrated that 2.5 ng/ml and higher concentration of TNFα significantly increased cell number/dish (Fig 1C). The most effective concentration was 25 ng/ml in donor 1 and 250 ng/ml in donor 2. TNFα did not affect colony number/dish. Cell number/colony was significantly higher at 2.5 ng/ml and and at higher concentrations of TNFα in both donors. Among 2.5, 25, 250 ng/ml TNFα, 25 ng/ml TNFα was selected for further analyses.

### Effect of 25 ng/ml TNFα on proliferation of synovial MSCs

**Fig 2:**
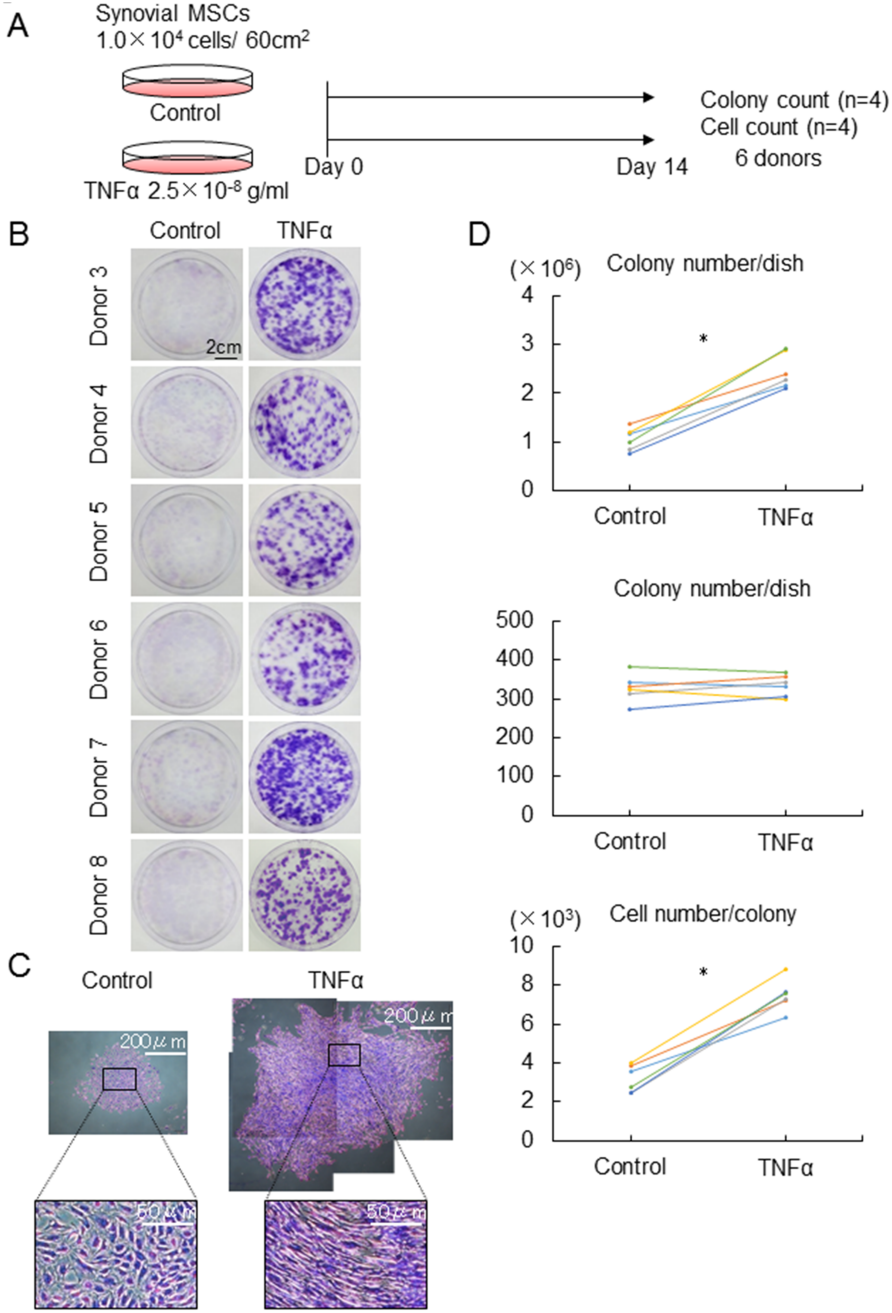
The effect of 25 ng/ml TNFα on proliferation of synovial MSCs. (A) Experimental design. Synovial MSCs were cultured with 2.5 x 10^-8^g/ml TNFα or without TNFα (Control) for 14 days. (B) Representative dishes stained with crystal violet (6 donors). (C) Representative cell colonies stained with crystal violet. Cell number/dish, colony number/dish, and cell number/colony. Average values are shown (n = 6, *p<0.05 by Wilcoxon signed-ranks test).

Next, the effect of 25 ng/ml TNFα on colony formation and proliferation of synovial MSCs was examined in the other 6 donors (Fig 2A). 25 ng/ml TNFα made colony formation more noticeable in all 6 donors (Fig 2B). The representative cell colony was larger and consisted of higher density of spindle shaped cells in synovial MSCs treated with TNFα (Fig 2C). Quantification analyses demonstrated that 25 ng/ml TNFα increased cell number/dish and cell number/colony in all 6 donors (Fig 2D). TNFα did not affect colony number/dish.

### Differentiation potentials of synovial MSCs pretreated with TNFα

**Fig 3:**
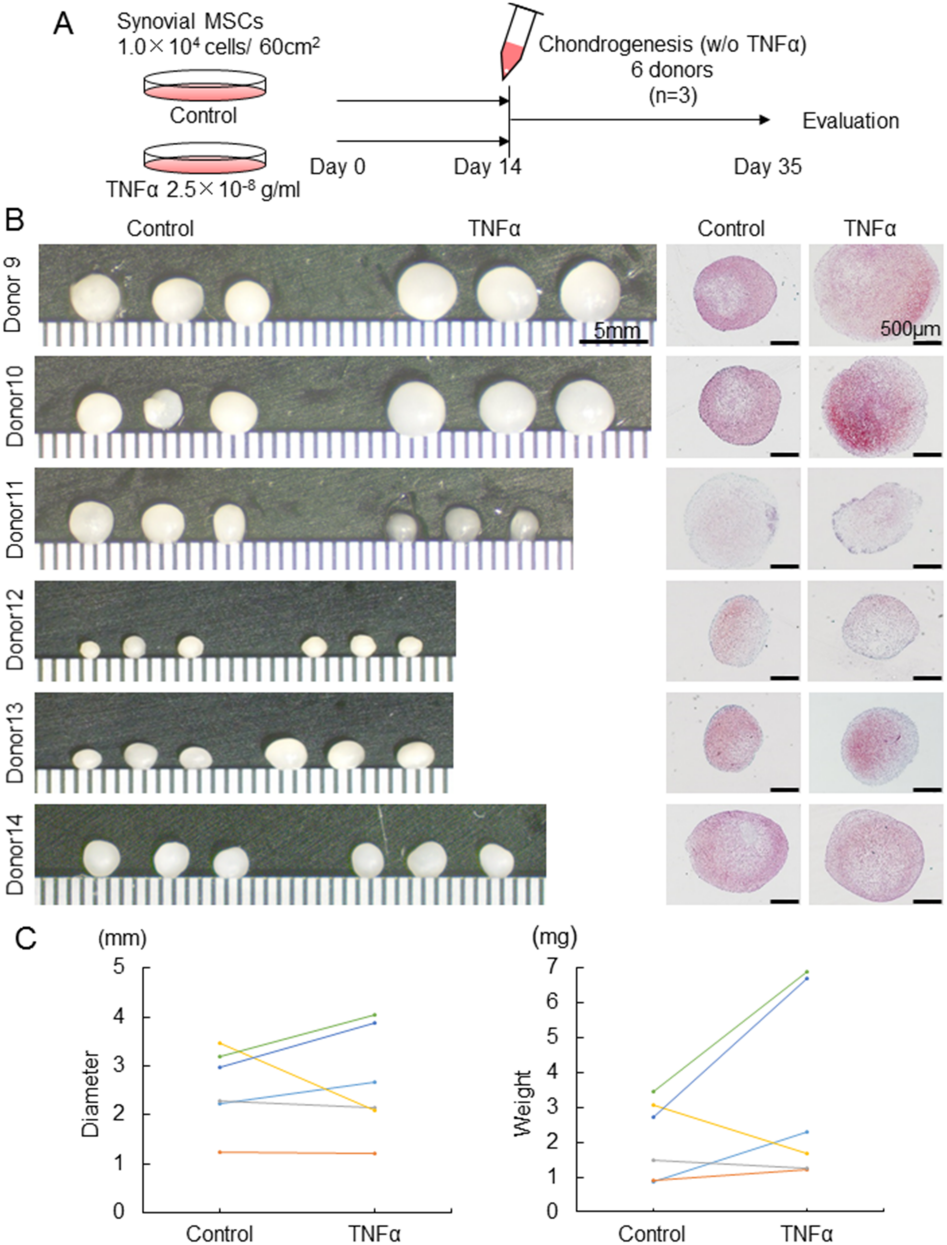
Comparison of chondrogenic potential of synovial MSCs pretreated with or without TNFα. (A) Experimental design. Synovial MSCs pretreated with 25 ng/ml TNFα or without TNFα (Control) were harvested, pelleted, and cultured in the chondrogenic medium without TNFα for 21 days. (B) Macroscopic and histological features of cartilage pellets. For histology, the sections were stained with safranin-o (C) Diameter and weight of the cartilage pellets. Average values are shown (n = 6, *p<0.05 by Wilcoxon signed-ranks test).

For comparison of chondrogenic potential of synovial MSCs pretreated with or without 25 ng/ml TNFα, those cells were pelleted and cultured in the chondrogenic medium without TNFα for 21 days (Fig 3A). Macroscopically and histologically, no obvious differences were shown between the control group and TNFα group, though some individual differences existed (Fig 3B). Quantification analyses showed no significant differences of diameter and weight of the cartilage pellets between both groups (Fig 3C). There were no significant differences in the amount of glycosaminoglycan (GAG) per pellet, DNA per pellet, and GAG /DNA of the cartilage pellets between the groups (S1 Fig).

**Fig 4:**
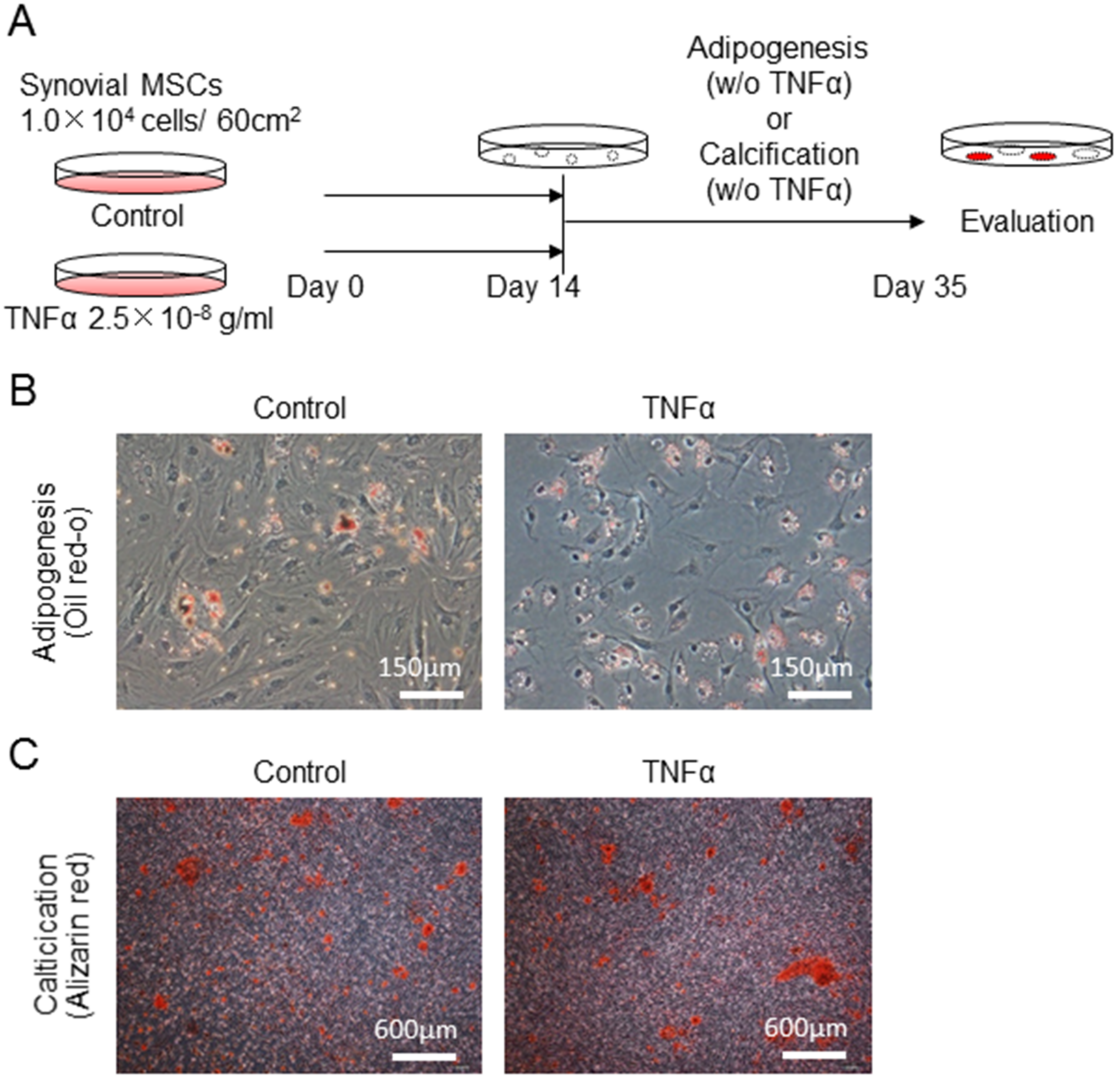
Comparison of adipogenic and calcification potential of synovial MSCs pretreated with or without TNFα. (A) Experimental design. Synovial MSCs were pretreated with 25 ng/ml TNFα or without TNFα (Control) for 14 days, then the medium was changed and the cells were cultured in adipogenic medium or calcification medium for further 21 days. (B) Representative cells stained with oil red-o for adipogenesis. (C) Representative cells stained with alizarin red for calcification.

For comparison of adipogenic and calcification potential of synovial MSCs pretreated with or without TNFα, those cells were cultured in the adipogenic and calcification medium without TNFα for 21 days (Fig 4A). No obvious differences were shown between the control group and TNFα group for adipogenesis (Fig 4B) and calcification (Fig 4C).

### Surface antigen expression of synovial MSCs treated with TNFα

**Fig 5:**
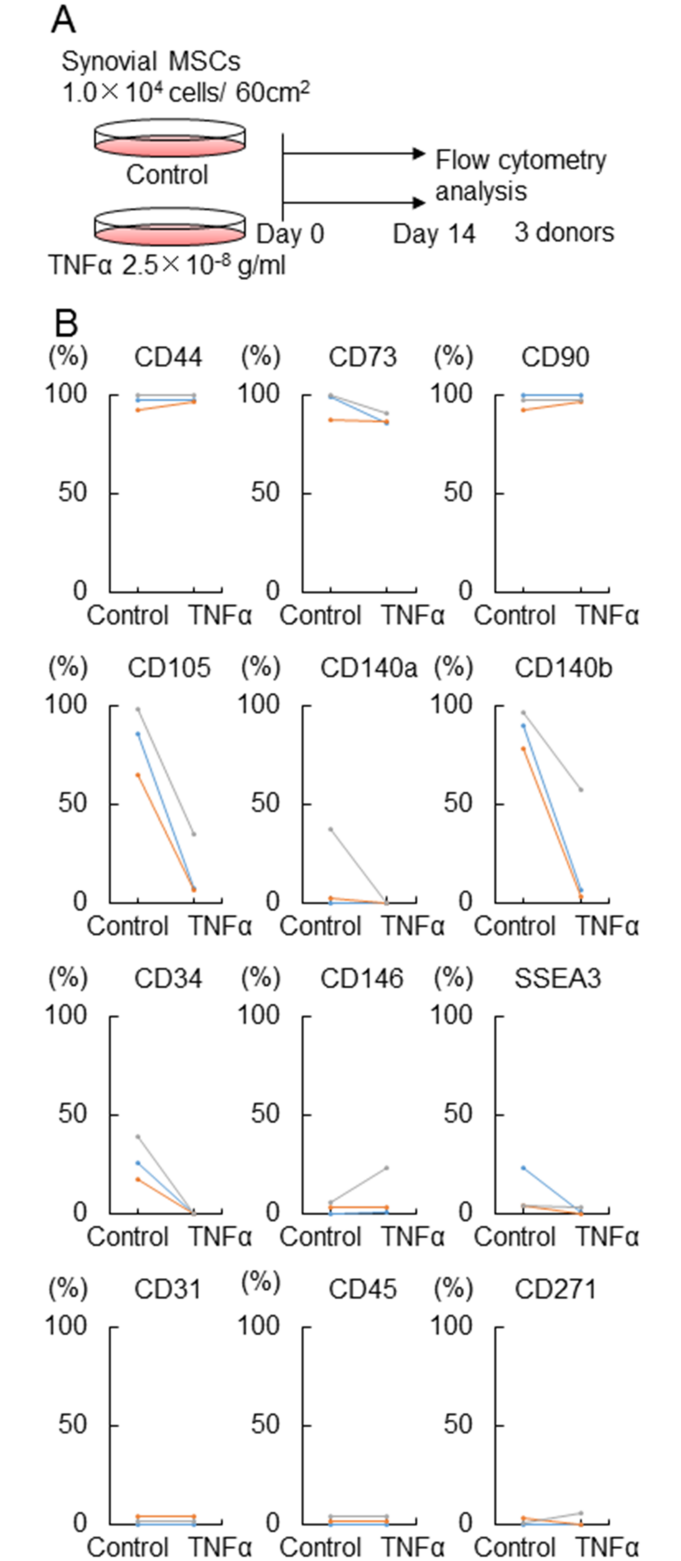
Surface antigen expression of synovial MSCs treated with TNFα. (A) Experimental design. Synovial MSCs were cultured with or without TNFα (Control) for 14 days for flow cytometry analysis. (B) Surface epitope expression. Average values are shown (n = 3).

For surface antigen expression, synovial MSCs were cultured with or without 25 ng/ml TNFα for 14 days and analyzed by flow cytometry in 3 donors (Fig 5A). TNFα did not affect expression rate of CD44, 73, and 90, while TNFα obviously decreased expression rate of CD 105 and 140b in all 3 donors. Expression rates of the other markers that we examined were relatively low in the cells irrespective of treatment of TNFα (Fig 5B).

## Discussion

In this study, we demonstrated that TNFα promoted proliferation of synovial MSCs. TNFα did not increase colony number but increased cell number/colony. Pretreatment with TNFα did not affect chondrogenesis of synovial MSCs. This is the first report showing the effect of TNFα on synovial MSCs.

As for the mechanism of how TNFα proliferated synovial MSCs, we speculated as follows according to the previous reports. TNFα stimulates the NF-kB pathway as a key regulator [16–18]. Though NF-κB is inhibited by IkB in a steady state, on the stimulation of TNFα, IkB is phosphorylated and the inhibitory effect of IkB decreases, which leads to the activation of the NF-κB pathway [19, 20]. NF-κB also regulates cyclin D1, which induces the cell cycle [21, 22].

In our study, 2.5 ng/ml and the higher concentration of TNFα significantly promoted proliferation of synovial MSCs. According to the previous reports in which the proliferative effect of TNFα was examined, the effective concentration was 25 ng/ml in synovial fibroblasts [13], 10 ng/ml in bone marrow MSCs [23], 4–10 ng/ml in neural stem cells [24], 10 ng/ml in cardiac fibroblasts [25], and 25 ng/ml in leiomyoma cells [26], The effective concentration of TNFα for those cells was similar or close to ours.

We demonstrated that TNFα increased cell number/colony. TNFα obviously increased the size and the cell density of the colonies. As far as we investigated, the effects of TNFα on cell colonies were not mentioned at all in the previous reports.

To calculate cell number/colony, both cell number/dish and colony number/dish could not be counted simultaneously in the same dish. Therefore, cell number/dish was counted from 4 dishes (dish A, dish B, dish C, dish D), and colony number/dish were counted from another 4 dishes (dish E, dish F, dish G, dish H). Then, cell number of dish A/colony number of dish E, cell number of dish B/colony number of dish F, cell number of dish C/colony number of dish G, cell number of dish D/colony number of dish H were calculated respectively. Finally, average and standard deviation in cell number/colony (n = 4) were determined. We were concerned about combination-dependent variability in cell number/colony, therefore we examined all of the combinations of dishes for Donor 1 for the case of 2.5 x 10^−8^ g/ml TNFα (Fig 1C). Using each combination the number remained 24 (= _4_P_4_), and both the average number and standard deviation in each combination were similar (S2 Fig).

In this study, TNFα did not affect colony number, indicating that TNFα did not affect the survival rate of synovial MSCs after adhesion. Although Wolfgang et al. reported that TNFα activated the expression of adhesion molecules such as vascular cell adhesion molecule-1 (VCAM-1) and migration [23], our experiments indicated that TNFα did not change the adhesive capacity of synovial MSCs to dishes that we used.

In our experimental conditions, the colony forming rate was quite low. This is because initial plating cell density was high. We plated 10,000 cells/dish because we required sufficient numbers of cells to examine the chondrogenic potentials of the MSCs. According to our previous reports, the colony forming rate of synovial MSCs decreased with higher initial cell seeding density due to colony-to-colony contact inhibition [2, 27, 28]. If the initial cell density was lower, the colony forming rate would have increased similar to our previous reports.

Pretreatment with TNFα did not affect chondrogenesis of synovial MSCs. Wehling et al. reported that the chondrogenic differentiation of bone marrow MSCs by pellet formation was inhibited by 10 ng/ml TNFα involving the NF-κB pathway [14]. On the other hand, Michal et al. reported that 10 ng/ml TNFα did not impair the chondrogenic differentiation of bone marrow MSCs by 3D high-density culture [15]. For these studies dealing with bone marrow MSCs, TNFα was added into chondrogenic medium, while for our current study, synovial MSCs were pretreated with TNFα and TNFα was not added in the chondrogenic medium. Therefore, the two methods with bone marrow MSCs are totally different from ours.

TNFα did not affect expression rate of CD44, 73, and 90, while TNFα obviously decreased expression rate of CD 105 and 140b in all 3 donors. CD105, also known as endoglin, is a type I membrane glycoprotein located on cell surfaces and is part of the TGFβ receptor complex. Fan et al. recently reported that the CD105 positive subpopulation of synovial MSCs had higher gene expressions of aggrecan, type II collagen and Sox9 as compared to those in CD105 negative cells [29]. This result was different from ours because their and our methods are different.

CD140b is known as PDGF receptor β. The PDGF family consists of 5 different isoforms, 4 homodimers (AA, BB, CC, DD) and 1 heterodimer (AB). Also, PDGF receptors consist of 2 homodimers (α/α, β/β) and 1 heterodimer (α/β). PDGF-AA activates only PDGF receptor α/α, PDGF-AB activates both PDGF receptor α/α and PDGF receptor α/β, and PDGF-BB activates all PDGF receptors [30]. These suggest that decreased expression of PDGF receptor β may lead to inhibition of proliferation of synovial MSCs through the PDGF signaling system, but the results were opposite. The TNFα signaling system overcame the decrease expression of PDGF receptor β for proliferation of synovial MSCs.

In our surface antigen analyses, the expression of CD34 was around 25% in synovial MSCs in the control group, though CD34 is known as a hematopoietic surface marker. The International Society for Cellular Therapy (ISCT) has proposed a set of standards to define MSCs, one of which was that the expression of CD34 was less than 2% [31]. In this statement, “these criteria will probably require modification as new knowledge unfolds” was noted, and there are recently increasing reports of CD34 positive MSCs derived from adipose tissue [32]. According to Lin et al., MSCs derived from adipose tissue are generally positive for CD34, and CD34 expression is lost in culture because CD34 is localized to the intima and adventitia of blood vessels in adipose tissue [33]. The expression of CD34 in synovial MSCs has not been fully investigated. We previously divided synovial tissue samples into 2 parts, one for histologic assessment and the other for the isolation of synovial MSCs. The number of blood vessels, by histological analysis, were correlated with the number of synovial MSCs, loosely indicating that one of the origins for synovial MSCs was blood vessels [34]. This concept was also advocated by Caplan et al [35]. These suggests that CD34 positive synovial cells can be regarded as MSCs in our current study.

In this study, we propose three limitations. Firstly, we do not know whether the addition of TNFα into the chondrogenic induction medium affects the chondrogenic potential of synovial MSCs, because TNFα was removed during chondrogenic induction. We can only conclude that pretreatment with TNFα did not affect the chondrogenic ability of synovial MSCs.

Secondly, our chondrogenic medium contained 1 μg/ml BMP7 which could have affected the TNFα effect on the chondrogenic potential of synovial MSCs. In vitro cartilage formation by bone marrow MSCs was first described by Johnstone et al; the medium used in their studies contained 10 ng/ml TGFβ without BMPs [36]. However, intensive investigations have shown that TGFβ alone did not fully differentiate MSCs into cartilage. The addition of BMP6 increased the wet weight of cartilage pellets by 10-fold, and they were stained more extensively for proteoglycans [37, 38]. Also, BMP2 and BMP4 promoted in vitro cartilage formation by bone marrow MSCs [39]. Furthermore, in an examination of the optimal combination of cytokines for in vitro cartilage formation by synovial MSCs, the combination of BMP2 and TGFβ was demonstrated to be the best among those assessed [40]. These findings indicate that TGFβ alone is not sufficient to differentiate MSCs into fully mature cartilage. In our current study we used BMP7 due to its availability in our laboratory. We previously examined the dose effect of BMP7 on in vitro cartilage formation of synovial MSCs and 1 μg/ml BMP7 formed the largest cartilage pellets, therefore, 1μg/ml BMP7 was used in this study as well as in other such studies [7, 27, 41, 42].

Thirdly, for clinical applications, TNFα could be useful because it is able to promote the proliferation of synovial MSCs and does not affect chondrogenesis. However, we found that TNFα decreased the surface expression of CD 105 and 140b, indicating that TNFα may affect some less obvious properties of MSCs. TNFα is known as an inflammatory cytokine, which is enriched in rheumatic joints, and induces cell apoptosis and tissue degeneration. The TNFα level in the synovial fluid of rheumatoid arthritis patients is around 300 pg/ml [43]. In our study, to increase the proliferation of synovial MSCs, TNFα is effective at 25 ng/ml, which is approximately 100 times higher than in the synovial fluid of rheumatoid arthritis patients. Before its clinical application, we will require further investigations as to the effectiveness and safety of synovial MSCs pretreated by TNFα for cartilage and meniscus regeneration.

In conclusion, TNFα promoted proliferation of synovial MSCs while maintaining chondrogenic differentiation potential.

## Supporting information

S1 Table Antibodies employed in this experiment. (TIF)

S1 Fig Comparison of glycosaminoglycan (GAG) and DNA of synovial MSCs pretreated with or without TNFα. (A) Experimental design. Synovial MSCs pretreated with 25 ng/ml TNFα or without TNFα (Control) were harvested, pelleted, and cultured in the chondrogenic medium without TNFα for 21 days, then GAG and DNA were evaluated. (B) GAG/pellet, DNA/pellet, GAG/DNA. Average values with standard derivation are shown (n = 6). There were no significant differences between two experimental conditions.
(TIF)

S2 Fig Calculation of cell number/colony. Cell number/colony in each possible combination. To calculate cell number/colony, cell number/dish was counted from 4 dishes (dish A, dish B, dish C, dish D), and colony number/dish was counted from another 4 dishes (dish F, dish G, dish H, dish I). Then, the cell number of dish A/colony number of dish F, cell number of dish B/colony number of dish G, cell number of dish C/colony number of dish H, and cell number of dish D/colony number of dish I were calculated respectively. Finally, the average and standard deviation in cell number/colony (n = 4) was determined. To examine the combination-dependent variability in cell number/colony, all of the combinations of dishes in Donor 1 for 2.5 x 10^−8^ g/ml TNFα (Fig 1C) were calculated.
(TIF)
