# Supplementary figures and images for: TNFα promotes proliferation of human synovial MSCs while maintaining chondrogenic potential
Source: PLoS One. 2017 May 18;12(5):e0177771. doi: 10.1371/journal.pone.0177771 (PMC5461123; doi:10.1371/journal.pone.0177771)

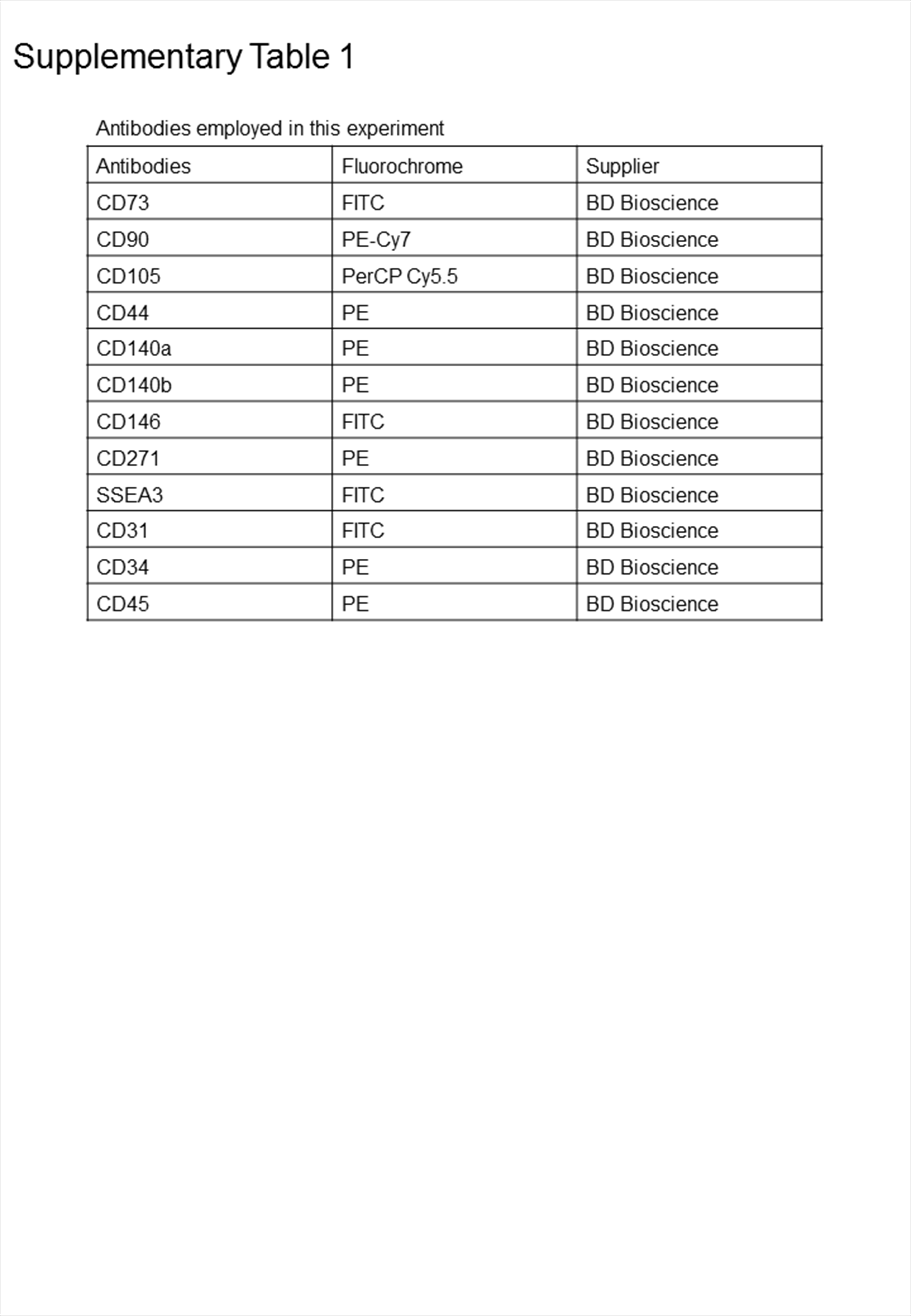

Supplement: S1 Table — (TIF) [file pone.0177771.s001.tif]

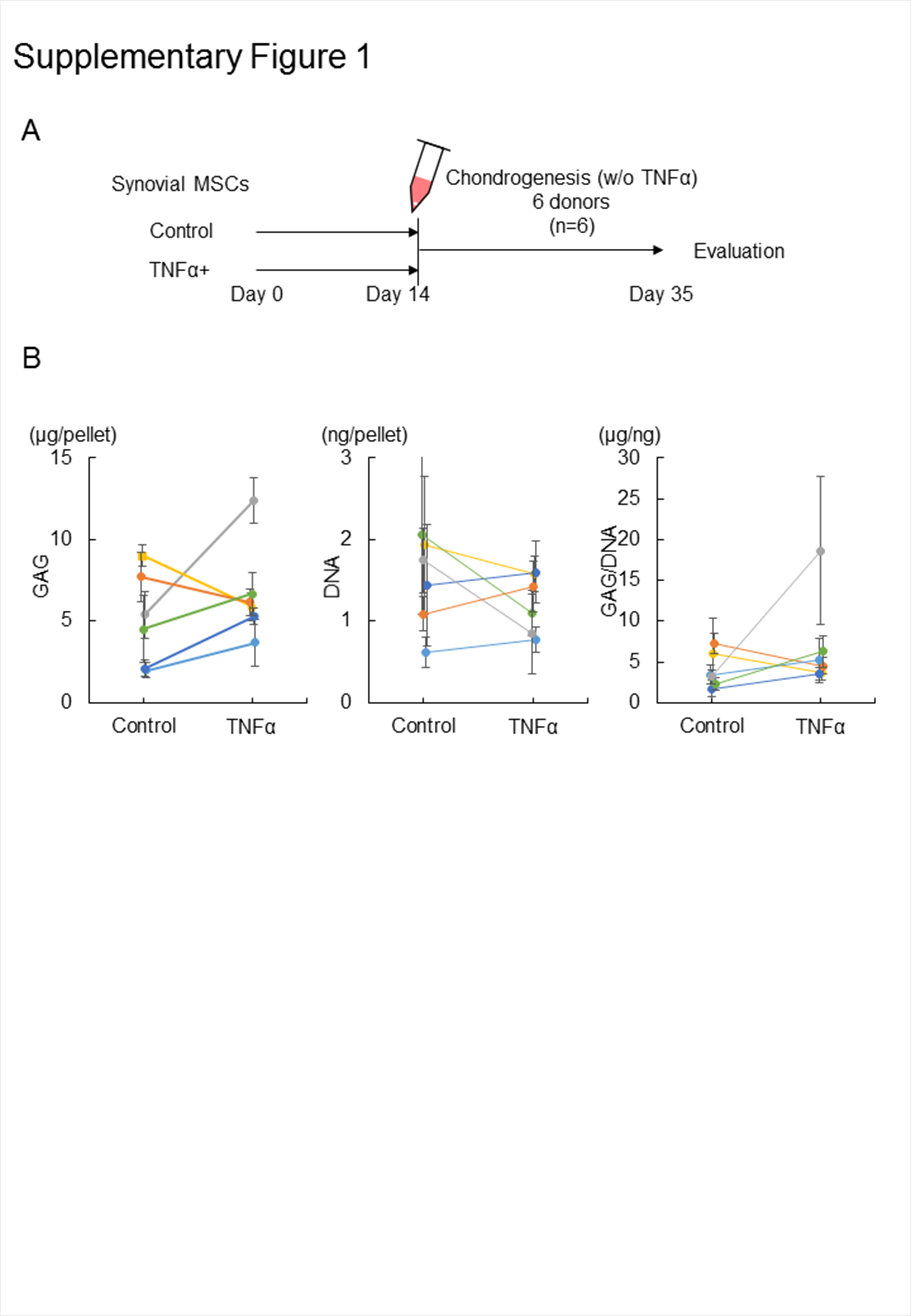

Supplement: S1 Fig — (A) Experimental design. Synovial MSCs pretreated with 25 ng/ml TNFα or without TNFα (Control) were harvested, pelleted, and cultured in the chondrogenic medium without TNFα for 21 days, then GAG and DNA were evaluated. (B) GAG/pellet, DNA/pellet, GAG/DNA. Average values with standard derivation are shown (n = 6). There were no significant differences between two experimental conditions. (TIF) [file pone.0177771.s002.tif]

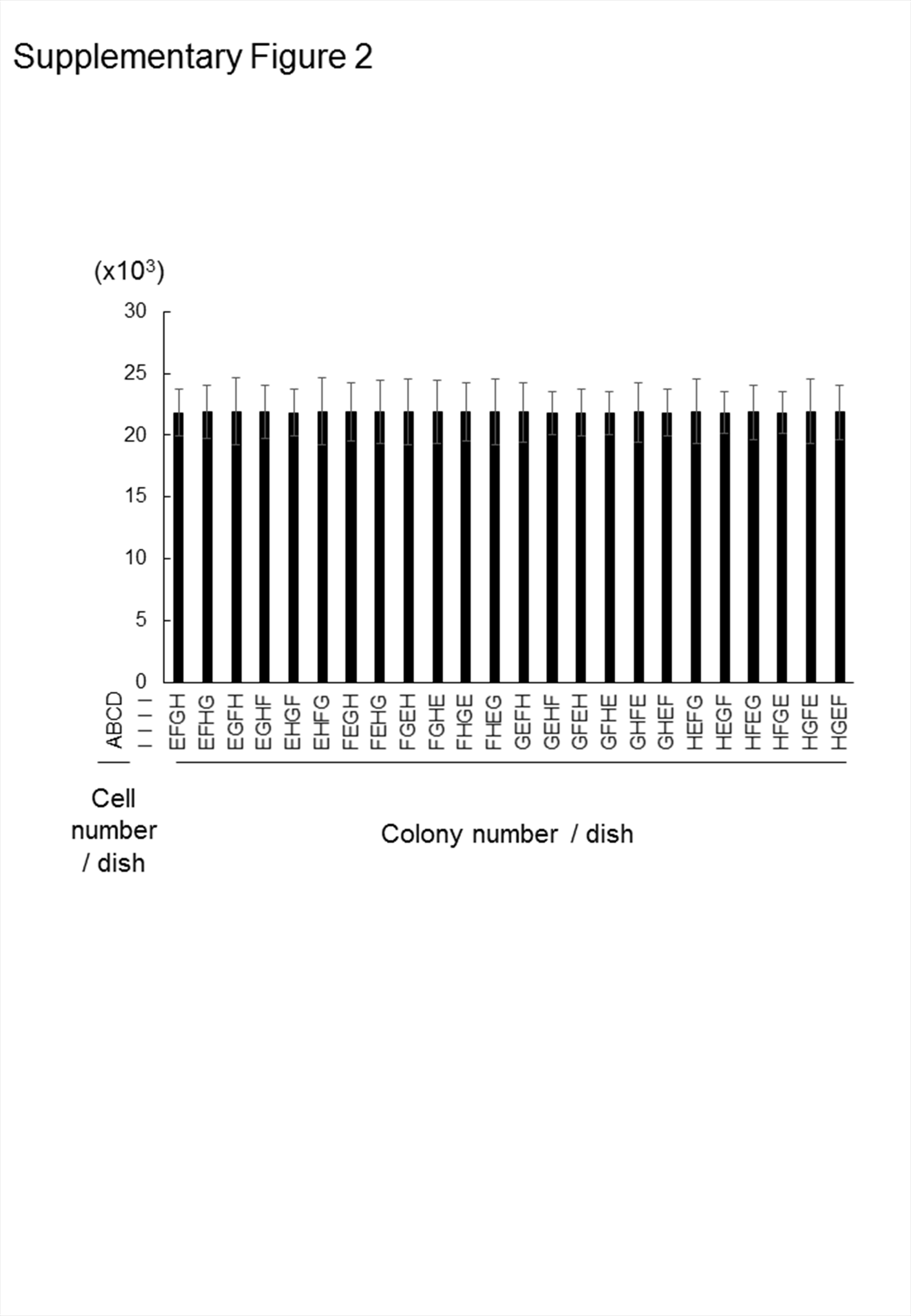

Supplement: S2 Fig — Cell number/colony in each possible combination. To calculate cell number/colony, cell number/dish was counted from 4 dishes (dish A, dish B, dish C, dish D), and colony number/dish was counted from another 4 dishes (dish F, dish G, dish H, dish I). Then, the cell number of dish A/colony number of dish F, cell number of dish B/colony number of dish G, cell number of dish C/colony number of dish H, and cell number of dish D/colony number of dish I were calculated respectively. Finally, the average and standard deviation in cell number/colony (n = 4) was determined. To examine the combination-dependent variability in cell number/colony, all of the combinations of dishes in Donor 1 for 2.5 x 10−8 g/ml TNFα (Fig 1C) were calculated. (TIF) [file pone.0177771.s003.tif]
